# Cellular engagement and interaction in the tumor microenvironment predict non-response to PD-1/PD-L1 inhibitors in metastatic non-small cell lung cancer

**DOI:** 10.1038/s41598-022-13236-8

**Published:** 2022-05-31

**Authors:** Angel Qin, Fatima Lima, Samantha Bell, Gregory P. Kalemkerian, Bryan J. Schneider, Nithya Ramnath, Madelyn Lew, Santhoshi Krishnan, Shariq Mohammed, Arvind Rao, Timothy L. Frankel

**Affiliations:** 1 Department of Internal Medicine, Division of Hematology-Oncology, University of Michigan, Ann Arbor, MI USA; 2 Department of Surgery, University of Michigan, 1500 East Medical Center Drive, Ann Arbor, MI USA; 3 Detroit Health Department, Detroit, MI USA; 4 VA Ann Arbor Healthcare System, Ann Arbor, MI USA; 5 Department of Pathology, University of Michigan, Ann Arbor, MI USA; 6 Department of Computational Medicine and Bioinformatics, University of Michigan, Ann Arbor, MI USA; 7 Department of Radiation Oncology, University of Michigan, Ann Arbor, MI USA

**Keywords:** Cancer microenvironment, Lung cancer

## Abstract

Immune checkpoint inhibitors (ICI) with anti-PD-1/PD-L1 agents have improved the survival of patients with metastatic non-small cell lung cancer (mNSCLC). Tumor PD-L1 expression is an imperfect biomarker as it does not capture the complex interactions between constituents of the tumor microenvironment (TME). Using multiplex fluorescent immunohistochemistry (mfIHC), we modeled the TME to study the influence of cellular distribution and engagement on response to ICI in mNSCLC. We performed mfIHC on pretreatment tissue from patients with mNSCLC who received ICI. We used primary antibodies against CD3, CD8, CD163, PD-L1, pancytokeratin, and FOXP3; simple and complex phenotyping as well as spatial analyses was performed. We analyzed 68 distinct samples from 52 patients with mNSCLC. Patients were 39–79 years old (median 67); 44% were male and 75% had adenocarcinoma histology. The most used ICI was atezolizumab (48%). The percentage of PD-L1 positive epithelial tumor cells (EC), degree of cytotoxic T lymphocyte (CTL) engagement with EC, and degree of CTL engagement with helper T lymphocytes (HTL) were significantly lower in non-responders versus responders (*p* = 0.0163, *p* = 0.0026 and *p* = 0.0006, respectively). The combination of these 3 characteristics generated the best sensitivity and specificity to predict non-response to ICI and was also associated with shortened overall survival (*p* = 0.0271). The combination of low CTL engagement with EC and HTL along with low expression of EC PD-L1 represents a state of impaired endogenous immune reactivity. Together, they more precisely identified non-responders to ICI compared to PD-L1 alone and illustrate the importance of cellular interactions in the TME.

## Introduction

Monoclonal antibodies against PD-1 (programmed cell death protein-1) and PD-L1 (programmed death-ligand 1) have changed the treatment landscape of metastatic non-small cell lung cancer (mNSCLC), which remains the number one cause of cancer-related mortality in both men and women^1^. These immune checkpoint inhibitors (ICI) are now approved as single agent therapy^2,3^ or in combination with cytotoxic chemotherapy^4,5^ as frontline treatment, after demonstrating a survival benefit compared to chemotherapy alone.

Though durable and impressive responses can be seen with ICIs, they are not without toxicity, with 25–35% of patients experiencing some type of immune-related adverse event (irAE) and 10–15% experiencing serious high-grade irAEs^6^. Furthermore, the response to ICI in mNSCLC with high PD-L1 expression ranges from ~ 45% as a single agent to ~ 60% in combination with chemotherapy, meaning a significant proportion of patients will not benefit from these therapies but are exposed to the potential toxicities.

A considerable amount of research has been dedicated to finding a predictive biomarker for ICI in mNSCLC. To date, the only accepted biomarker is tumor expression of PD-L1, represented as a tumor proportion score (TPS), with a score ≥ 50% indicating a high likelihood of response. However, PD-L1 TPS is an imperfect biomarker as its predictive significance varies among different approved PD-1/PD-L1 inhibitors. Furthermore, there is both intratumoral heterogeneity of PD-L1 expression^7^ and differential expression based on disease site (lung, lymph node, distant metastasis)^8^ which further complicates the predictive significance of a single PD-L1 TPS. The focus thus far has been on predictors of response. Equally important is elucidating characteristics that are associated with or predict lack of response.

It is increasingly recognized that the non-neoplastic constituents of the tumor microenvironment (TME) such as fibroblasts and various immune cells play a key role in tumor progression and metastasis as well as response to immune-based therapies^9^. Galon et al.^10^ performed immunostaining on surgically resected, early stage colon cancers and found that the density of specific T cells was associated with prognosis; specifically patients with low densities of CD3^+^ and CD45RO^+^ memory T cells had the same adverse prognosis as patients with metastatic disease.

The most frequently used methods to investigate the immune TME include traditional immunohistochemistry (IHC) and flow cytometry. The former suffers from limitations in the number of epitopes probed per slide making multiantigen phenotyping difficult, while the latter is performed on a single cell suspension, ignoring valuable spatial information. Multiplex fluorescence immunohistochemistry (mfIHC)^11^ uses signal amplification and in situ fluorescent staining to provide accurate multiantigen phenotyping while preserving spatial relationships. Currently available staining and imaging platforms, paired with analysis software, provide a potential valuable clinical method for automated TME description.

To study the impact of immune cell interactions on response and non-response to ICI, we performed mfIHC on tissue from patients with mNSCLC who received ICI and were followed for treatment response. Using phenotypic and spatial data, we characterized the interactions of components of the TME and evaluated the ability to predict response to ICI therapy.

## Materials and methods

### Patients

This was a retrospective study approved by the Institutional Review Boards of the University of Michigan and Ann Arbor VA. The need for informed consent was waived by the University of Michigan Office of Ethics, Integrity, and Compliance and the Ann Arbor VA Ethics Committee due to the retrospective nature of the study. All patients had a diagnosis of metastatic NSCLC and were treated with standard of care immunotherapy between 2015 and 2017 at one of the two institutions. Tissue from routine, diagnostic biopsies was used for all analyses. All research was conducted in accordance with all guidelines and regulations set forth by both institutions.

### Multiplex fluorescent immunohistochemistry staining and imaging

Our methods have been previously published and validated^12,13^. Briefly, slides of whole tissue were subjected to deparaffinization with xylene for 10 min. The Opal 7 manual kit (Akoya Biosciences) was used according to the manufacturer’s instructions. After each antigen retrieval, slides were stained with antigen-specific primary antibodies followed by Opal Polymer (horseradish peroxidase (HRP) conjugated secondary antibody). Application of the Opal tyramide signal amplification (TSA) reagent created a covalent bond between the fluorophore and the tissue at the site of the HRP attached to the secondary antibody. Each antigen retrieval step was performed using either AR6 or AR9 antigen retrieval buffer, which allowed for the removal of prior primary and secondary antibody while the fluorophore remained covalently bound to the tissue antigen. Validation that subsequent antigen retrieval steps removed the prior primary antibody has been reported^14^. See Supplemental Table 1 for antibodies and dilutions. Imaging was performed using the Mantra Quantitative Pathology Work Station. Multiple images per sample were captured at 20× magnification and used for further analysis. Individual images were analyzed independently and a weighted mean value determined for each patient.

### Image analysis

Image analysis was conducted using the same methods as our previously published studies^12,13^. In brief, images were analyzed using inForm Cell Analysis software (Akoya Biosciences). Using the inForm training software, both tissue and cell compartments were identified and segmented. Tissue was segmented into stroma and epithelial cancer compartments, while cells were segmented into nucleus, cytoplasm, and membrane compartments. DAPI stain was used to determine the size and shape of each nucleus, and an x- and y- coordinate was assigned to each cell. The cytoplasmic shape, thickness, and distance from the nucleus were calculated by the automated system using fluorescence of CD8, CD163, and CD3. Each pixel on the image represented 0.496 microns.

After cell segmentation, the following cells were phenotyped using the trainable software after select cells were manually assigned based on single staining criteria: cytotoxic T cells (CTL; CD3^+^ CD8^+^), helper T cells (HTL; CD3^+^ CD8^−^), regulatory T cells (Treg; CD3^+^, CD8^−^, FoxP3^+^), APCs (CD163^+^), epithelial tumor cell (ECs; pancytokeratin^+^), and other cells (CD3-, CD163-, pancytokeratin^−^). PD-L1 was evaluated on both APCs and ECs. After the fluorescent intensity score was determined for PD-L1, CD8, and FoxP3, R based software, in combination with the original cell phenotypes produced by inForm, was used to formulate multiantigen phenotypes. See Table 1 for the color schema of each phenotype. The nearest neighbor (nearest distance from one cell to another) and cell-to-cell engagement were calculated using methods that have been previously described^12^. In brief, the nearest neighbor was calculated as the intercellular distance using the assigned x- and y- coordinates. For engagement, a 15 μm radius circular area was selected around each T cell, and a 40 μm radius circular area was defined around APCs and ECs. The frequency of other cell types within these defined radii were calculated.

**Table 1:** Cellular phenotype and color.

Phenotype	Markers	Color
PD-L1^−^ EC	PD-L1^−^, Pancytokeratin^+^	White
PD-L1^+^ EC	PD-L1^+^, Pancytokeratin^+^	Light purple
HTL	CD3^+^, CD8^−^, FoxP3^−^	Green
CTL	CD3^+^, CD8^+^, FOXP3^−^	Yellow
Treg	CD3^+^, CD8^−^, FoxP3^+^	Red
PD-L1^−^ APC	CD163^+^, PD-L1^−^	Orange
PD-L1^+^ APC	CD163^+^, PD-L1^+^	Magenta

### Statistics

All statistics were performed using JMP 14 software, unless otherwise noted. These are also in concordance with our previously published studies^12,13^. Differences in phenotype, distances, and engagement were evaluated by 2-tailed Student’s t test or ANOVA. For data not normally distributed, nonparametric Wilcoxon rank-sum was used. Categorical variables were analyzed with Fisher’s exact test, and *p* ≤ 0.05 was considered significant. P values were adjusted for multiple testing using the Benjamini–Hochberg false discovery rate procedure. Statistical significance was then assessed via an adjusted *p* value of 0.05. For survival analysis, Kaplan–Meier plots were drawn and statistical differences were determined by log-rank.

### Ethics approval

This was a retrospective study approved by the IRBs of the University of Michigan and Ann Arbor VA.

### Consent to participate

As this was a retrospective study on patient material only, patient consent was not required; this was specified in the respective IRB approvals.

### Consent to publish

This is not applicable to this study as no individual data is showed.

## Results

### Patient characteristics

68 distinct samples from 52 patients with mNSCLC were used (n = 1269 total images). Patients were 39–79 years old (median 67); 44% were male and 75% had adenocarcinoma histology. Biopsies were obtained from the primary site (64%), metastatic LN (20%), and a distant metastatic site (16%). Fourteen patients had more than one sample analyzed—these samples were obtained at distinct time periods, i.e. at time of surgical resection and at time of development of metastatic disease, but all prior to treatment with ICI. The most common ICI was atezolizumab (48%) which was primarily given in the 2nd line setting; during this time, ICI was not approved for front-line use. Non-responders were defined as patients with a best response of PD (progressive disease), whereas responders were defined as those without PD (i.e. stable disease, partial response, and complete response) as defined by RECIST criteria, per the treating physician. 43% of patients were non-responders while 57% were responders to ICI. See Table 2 for a summary of their characteristics.

**Table 2:** Patient characteristics.

Characteristic	
Age at diagnosis (median, range)	65.5 (39–79)
**Sex (n, %)**	
Male	23 (44.2%)
Female	29 (55.8%)
**Smoking history (n, %)**	
Current	4 (7.7%)
Former	39 (75%)
Never	9 (17.3%)
**Histology (n, %)**	
Adenocarcinoma	39 (75%)
Squamous cell carcinoma	11 (21.2%)
Other	2 (3.8%)
**Lines of previous therapy**	
Mode (%), range	1 (38.5%), [0–5]
**Immunotherapy (n, %)**	
Atezolizumab	25 (48.1%)
Nivolumab	16 (30.8%)
Pembrolizumab	11 (21.2%)

### mfIHC confirms the importance of PD-L1 expression in predicting lack of response to therapy

**Figure 1:**
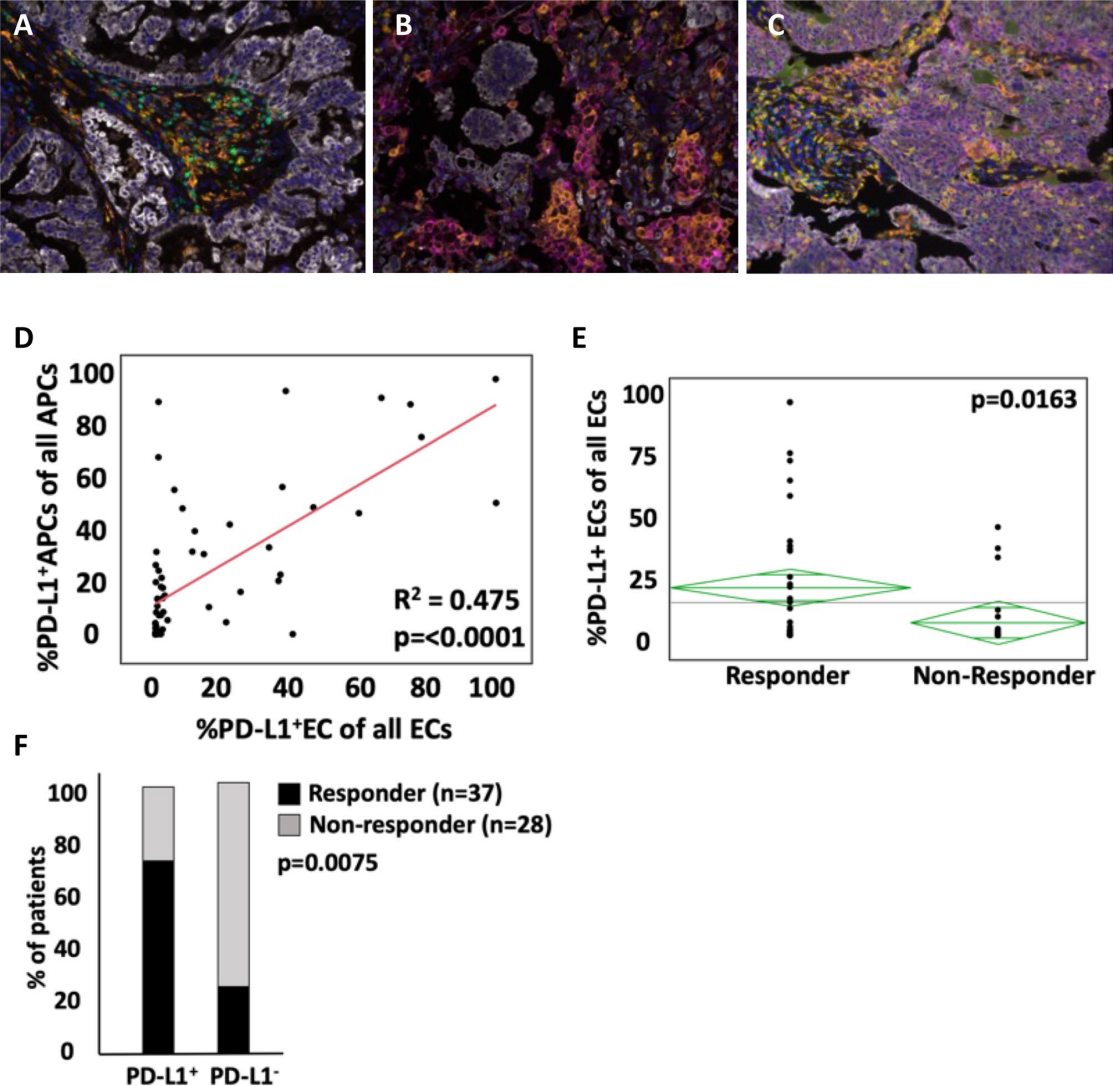
PD-L1 staining patterns on tumor cells and antigen presenting cells. 3 distinct patterns of PD-L1 expression are noted: (**A**) no PD-L1 expression (**B**) primary expression on APCs (magenta) and (**C**) primary expression on tumor cells (light purple). (**D**) positive correlation between APC and EC PD-L1 expression. (**E**) lower EC PD-L1 expression in non-responders compared to responders. (**F**) EC PD-L1 expression in responders and non-responders, with significantly more non-responders in the PD-L1^−^ group. All images are obtained at 20 × magnification.

We initially performed staining to show that mfIHC can accurately describe the immune cell infiltration of NSCLC (see Supplemental Figure 1). To determine if our staining and analysis platform recapitulated prior data linking PD-L1 to treatment efficacy, non-responder and responder tumors were stained for checkpoint surface expression. Three subsets of tumors were identified, including those with no PD-L1 expression (Fig. 1A), primary expression on APCs (magenta; Fig. 1B) and tumor cells (light purple; Fig. 1C). There was significant correlation between expression on both cell types (Fig. 1D; R^2^ = 0.475, *p* < 0.0001). To determine impact of PD-L1 expression on treatment response, patients were dichotomized to those with disease progression as their best response and those that had at least stable disease. Patients in the non-responder group had significantly lower mean PD-L1 TPS than responders (5.6% vs 20.6%; *p* = 0.0163) (Fig. 1E). When stratifying patients by total PD-L1 positivity in ECs and APCs, non-responders were more likely to be PD-L1 negative (54%) when compared to responders (24%) (Fig. 1F). Interestingly, although a majority of the patients with no PD-L1 expression were non-responders (n = 10/14), four patients with no PD-L1 expression responded to ICI therapy. Overall, these findings are in line with clinical observations in lung cancer where treatment decisions based on the TPS.

### Lack of APC and helper T cell engagement with cytotoxic T lymphocytes is associated with no ICI response

**Figure 2:**
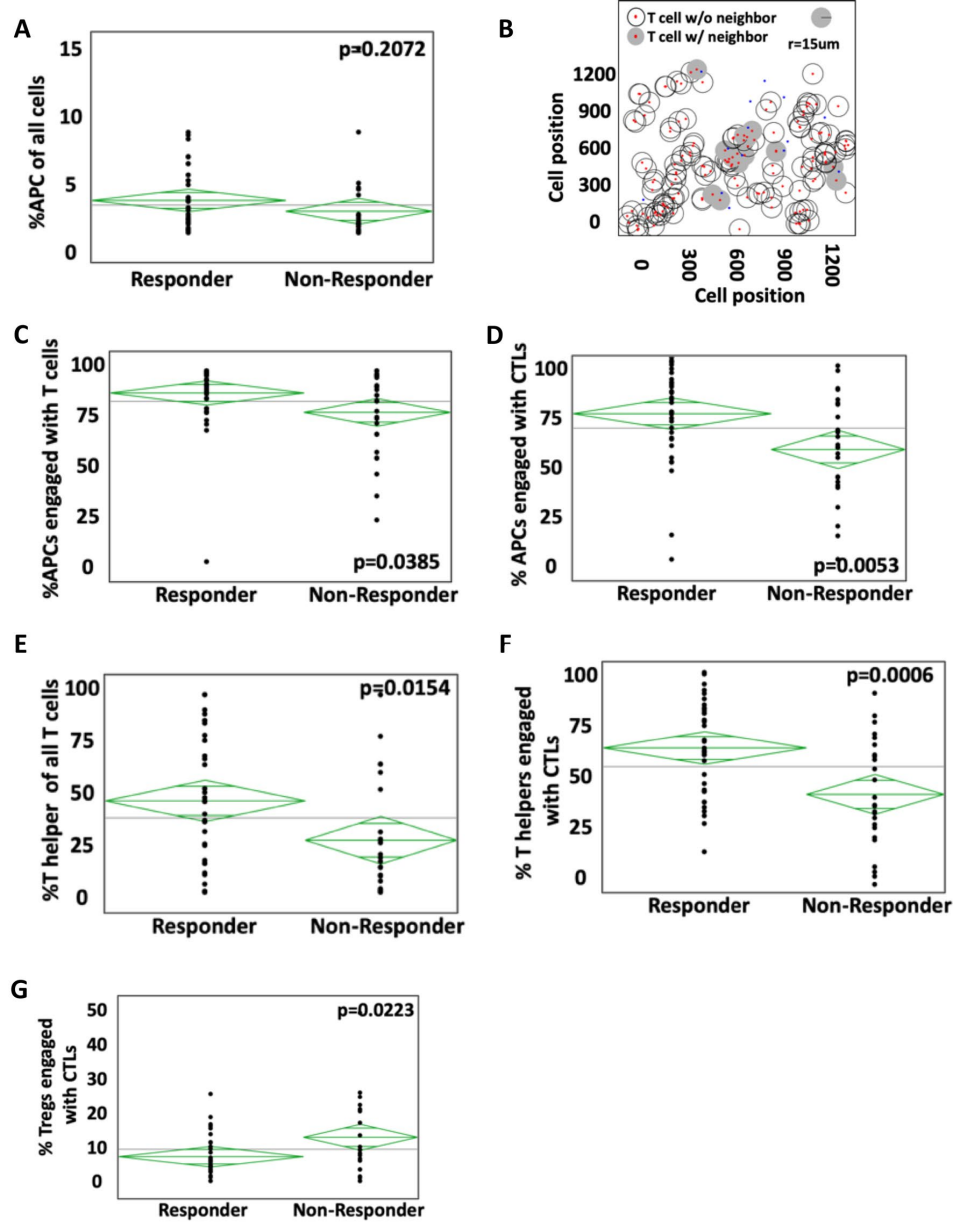
Interactions of antigen presenting cells (APCs) and helper T cells (HTLs) with cytotoxic T cells (CTLs) in responders and non-responders. (**A**) Percentage of all cells that are APCs in responders versus non responders. (**B**) Example of mathematical measurement of CTLs with and without neighbors, by using a 15 μm radius as defining engagement. (**C**) Percentage of APCs engaged with T cells in responders compared to non-responders. (**D**) Percentage of APCs engaged specifically with CTLs. (**E**) Percentage of all T cells that are HTLs in responders versus non responders. (**F**) Lower engagement of HTL to CTL in non-responders compared to responders. (**G**) Higher engagement of regulatory T cells (Tregs) with CTL in non-responders compared to responders.

A critical step in generating an immune response is priming of cytotoxic T cells by antigen presenting cells. To assess this in our samples, we analyzed the spatial relationships of both cell types in the TME with the assumption that increased incidence of cell contact may be associated with enhanced priming. We found no difference in the relative quantity of APCs between responders and non-responders (Fig. 2A). To determine if spatial proximity of cells was important, we measured engagement of APCs and CTLs in the TME. Using the 15 μm radius as a measurement of engagement, we were able to examine each CTL and whether it had a neighbor in graphical view (Fig. 2B). There was lower percentage of APCs engaged with T cells in non-responders (78.4%) when compared to responders (88.6%; *p* = 0.0385, Fig. 2C). This was particularly pronounced for CTLs where mean engaged APCs were 54.2% and 71.8% for non-responders and responders, respectively (*p* = 0.0053, Fig. 2D). These data confirm that decreased engagement of CTLs with APCs is associated poor response to checkpoint therapy in NSCLC.

While abundant data exist on the role of CD8^+^ CTLs in ICI therapy, little is known about helper T cells that augment the immune response through the release of pro-inflammatory cytokines such as IFNγ and TNF^15^. To study their association with response, the spatial location of HTLs in the TME was assessed relative to other cell types. ICI non-responders were found to have a lower percentage of HTLs of all T cells compared to responders (Fig. 2E). We next sought to measure the engagement of HTL with CTLs with the hypothesis that supportive cytokines may result in enhance tumoricidal capabilities. The percentage of HTLs engaged with CTLs was significantly lower in non-responders compared to responders (42.5% and 62.7%, respectively, *p* = 0.0006, Fig. 2F). To determine if this engagement was related to the presence of regulatory T cells, tissues were stained for the nuclear marker FoxP3. Non-responders were found to have significantly higher percentage of Tregs engaged with CTLs compared to responder (*p* = 0.0223, Fig. 2G).

### Differences in spatial relationships between cytotoxic T cell, tumor cells and helper T cells are observed in those who do not respond to ICI

**Figure 3:**
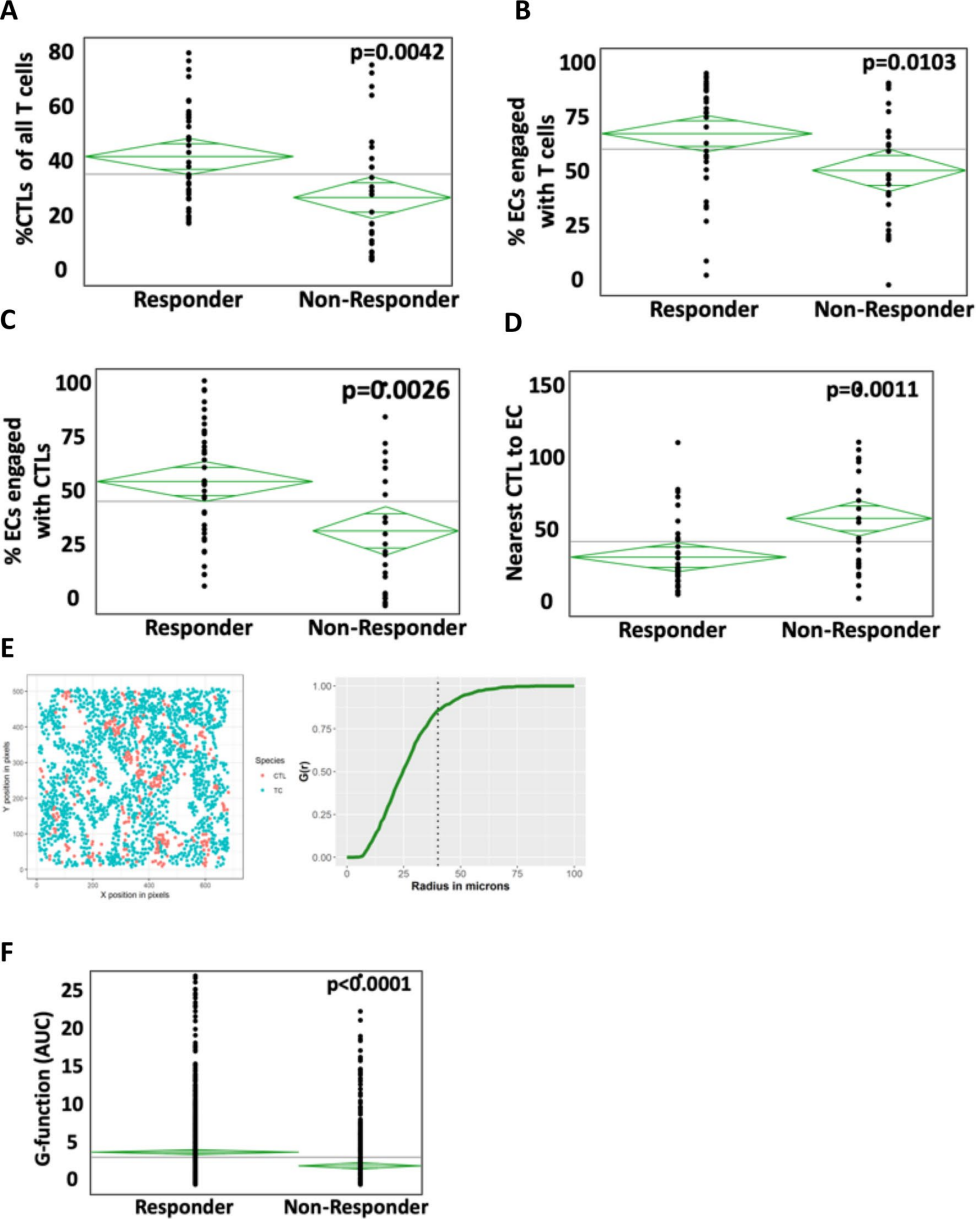
Engagement of tumor cells (ECs) with cytotoxic T cells (CTLs) in responders and non-responders. (**A**) Percentage of all T cells that are CTLs in responders versus non responders. (**B**) EC engagement with T cells and specifically with CTLs (**C**) in responders versus non-responders. (**D**) The distance between EC and nearest CTL is significant shorter in responders than non-responders. (**E**) Description of G-cross which is a mathematical representation of cellular mixing at a population level The degree of cellular mixing numerically represented as the as the AUC. (**F**) Cellular mixing, as defined by the AUC of the G-cross, is lower in non-responders compared to responders.

CTLs are thought to represent the primary effector of ICI therapy as antibodies directly interfere with inhibitory receptors on their surface. Total lymphocyte infiltration as well as spatial relationships to tumor and other immune cells in responders and non-responders was measured. Non-responders had a significantly lower proportion of CTLs of total T cells, 25.5% versus 42.1%, *p* = 0.0562 (Fig. 3A). Nearest neighbor and engagement analysis were used to determine if the increased infiltration of CTLs translated to greater proximity to tumor cells. Quantifying these relationships revealed that tumors not responding to ICI had less engagement between all T cells and ECs (60.7% vs 75.7%, *p* = 0.0103) which was particularly pronounced for CTLs to ECs (33.8% vs 54.1%, *p* = 0.0026) (Fig. 3B, C). When calculating total intercellular distance, the nearest CTL was significantly further away from tumor cells in non-responders compared to responders (Fig. 3D). To provide a higher level population based analysis, G-cross, a mathematical representation of cellular mixing at a population level^13,16^, was employed. In this model system, degree of cellular mixing for a given image is numerically represented as the area under the curve at a fixed radius from a graph of probability of intercellular distance such that a higher value denotes greater mixing (Fig. 3E). Application of g-cross to this patient cohort confirmed that CTLs and ECs were significantly less mixed in patient not responding to ICI therapy with *p* < 0.0001 (Fig. 3F).

### Spatial relationship of cells in addition to tumor PD-L1 distinguishes responders and non-responders

**Figure 4:**
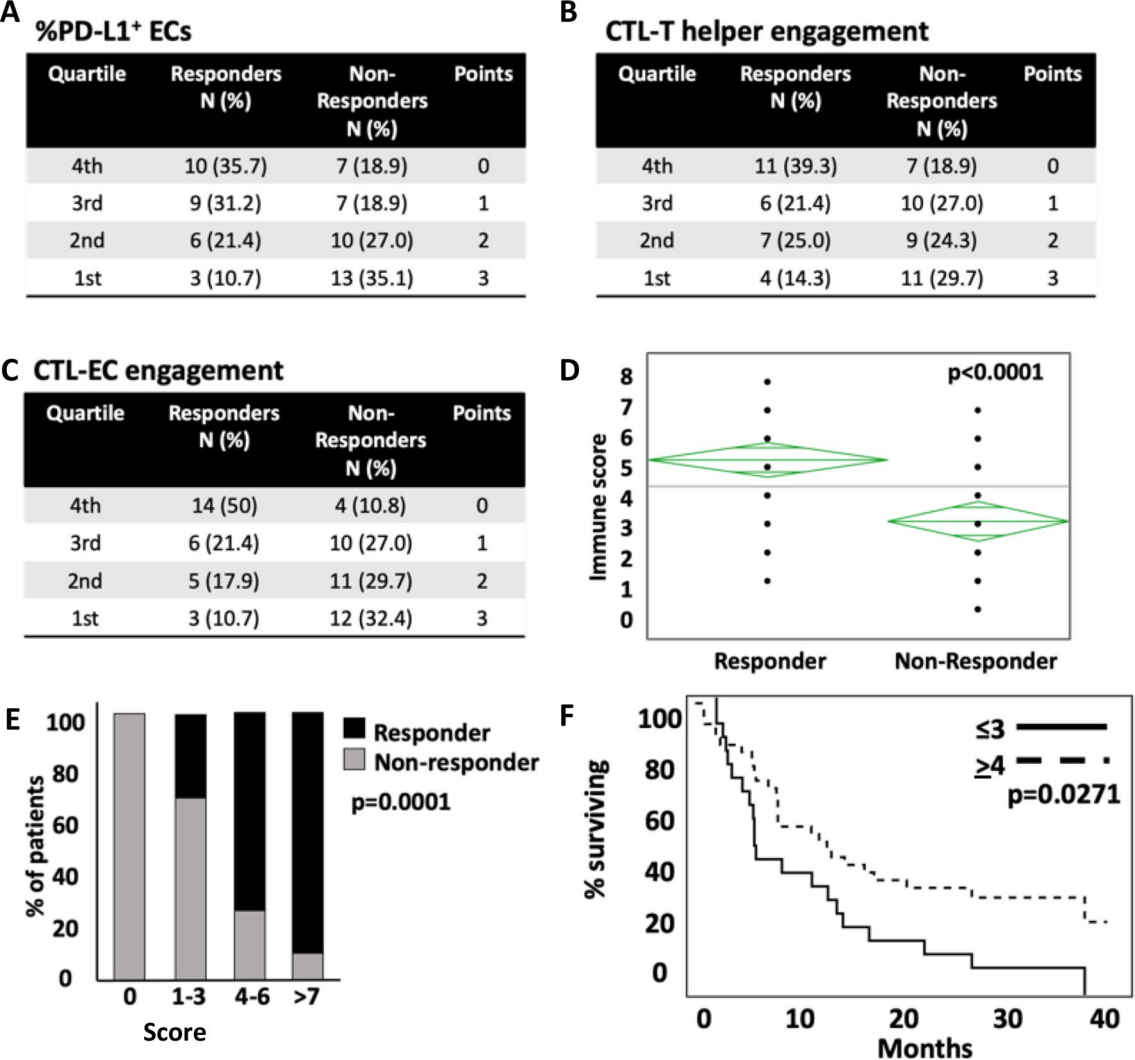
Combining spatial relationships with tumor PD-L1 to generate a spatial immune score to distinguish responders and non-responders to ICI. Each relationship, tumor PD-L1 (**A**), CTL-HTL engagement (**B**), and CTL-EC engagement (**C**) was divided into quartiles based on expression or degree of engagement. Each quartile was assigned a point, with 3 points assigned to the 1st quartile (representing greatest expression or engagement). The points were added up to generate the spatial immune score, with responders having a significantly higher score compared to non-responders (**D**). (**E**) Break down of spatial immune score between responders and non-responders. (**F**) All patients with zero points were non-responders.

Multivariate analysis was used to determine the factors most associated with non-response to ICI therapy. Each characteristic was divided into quartiles with the 1st quartile having the highest expression or engagement and the 4th quartile having the lowest expression or engagement. Each quartile was assigned a point, with the 1st quartile receiving 3 points and the 4th quartile receiving 0 points (Fig. 4A–C). Assuming a potential synergistic benefit of multiple factors in predicting treatment efficacy, an aggregate score, termed the “spatial immune score”, was calculated by combining PD-L1 expression, CTL–EC engagement, and CTL–HTL engagement. Non-responders had a statistically significant lower spatial immune score compared to responders (Fig. 4D) with all patients scoring 0 points not responding to therapy (Fig. 4E). Patients with a score ≤ 3 also had a statistically significant shorter survival when compared to those with a score ≥ 4 (Fig. 4F).

### Spatial data improves the predictive capability PD-L1 expression and identifies biologically unique subsets of NSCLC patients

**Figure 5:**
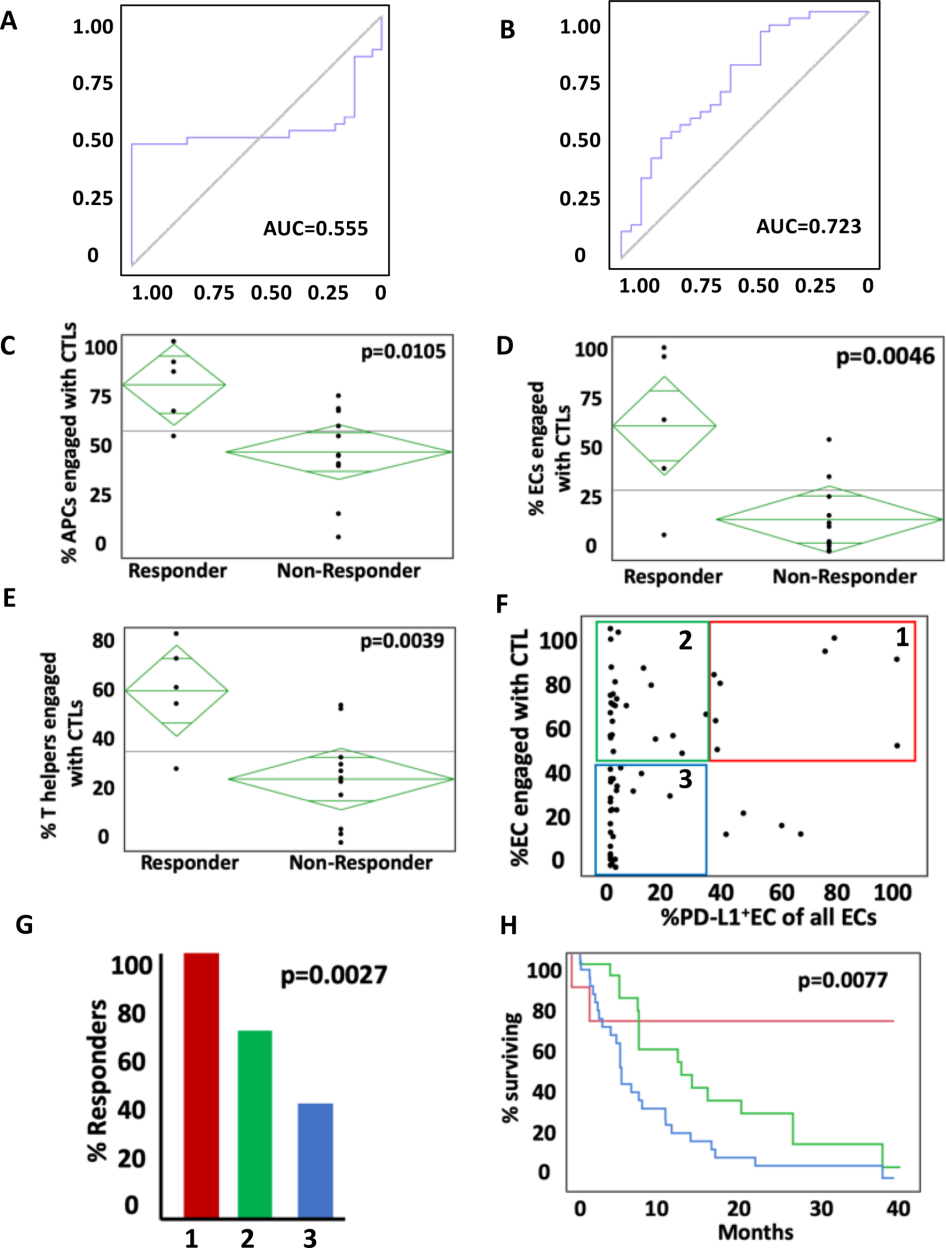
Predictive ability of spatial relationships in addition to tumor PD-L1 to predict non-response to ICI. (**A**) AUC of tumor PD-L1 alone to predict non-response versus (**B**) AUC of tumor PD-L1 and spatial relationships combined together. (**C**–**E**) Examination of 17 patients with PD-L1 < 1%, who are thought to not likely benefit from ICI. Even in the absence of PD-L1, engagement of CTLs with APCs (**C**), ECs (**D**), and HTLs (**E**) were able to separate responders from non-responders. (**F**) Examining the interplay between endogenous immune reactivity and checkpoint inhibition by dividing patients into 3 cohorts based on EC-CTL engagement and EC PD-L1 expression. (**G**) Cohort 1, which has high engagement and high PD-L1 expression likely representing reactive upregulation defined the patients that all had a response to ICI and also had the longest median overall survival (**H**).

To determine the predictive ability of spatial factors added to traditional PD-L1 expression, models were created which included PD-L1 alone or PD-L1 plus the previously described spatial metrics. When only PD-L1 expression was used, the calculated AUC as a predictor of non-response to ICI was 0.55 (Fig. 5A). When the spatial immune score components were added to this, the accuracy increased with an AUC of 0.72 (Fig. 5B).

To explore this further, we looked at patients who are not expected to respond to a PD-1/PD-L1 inhibitor, i.e. those with PD-L1 TPS < 1% (n = 17). Analysis of cellular spatial relationships revealed findings similar to the larger cohort. Tumors without PD-L1 expression were more likely to respond to treatment if CTLs were more actively engaged in the TME with APCs, tumor cells and HTLs (Fig. 5C–E).

The interplay between endogenous immune reactivity and checkpoint expression is a complex one. In some instances, PD-L1 is inherently expressed and prevents activation of neighboring CTLs. In other situations, checkpoints are expressed in response to immune activation, often mediated by interferon gamma, which serves to quell the CTL activity. To study this further in our patient cohort, subjects were divided into three groups based on their CTL-EC engagement and tumor PD-L1 expression: Group 1: high engagement and high PD-L1 expression representing reactive upregulation; Group 2: high engagement and low PD-L1 expression suggestive of a passive relationship between tumor cells and CTLs; Group 3: low engagement and low PD-L1 expression representing inherent immune resistance (Fig. 5F). Patients in group 1 universally responded to ICI therapy (Fig. 5G, *p* = 0.0027) and also had the longest survival (median survival not reached compared 14 months for group 2 and 6.5 months for group 3) (Fig. 5H, *p* = 0.0077). These data suggest that spatial relationships of immune and tumor cells can significantly add to the predictive capability of the currently used PD-L1 expression alone.

## Discussion

Immune checkpoint inhibitors against PD-1/PD-L1 have rapidly altered the treatment landscape of NSCLC. From the initial approval of nivolumab (PD-1 inhibitor) in 2015 for patients with metastatic NSCLC and progression after platinum-based chemotherapy, these agents are now approved as frontline treatment of metastatic NSCLC with or without chemotherapy, as consolidation therapy after concurrent chemotherapy and radiation in locally advanced NSCLC, and in the adjuvant setting after surgical resection and adjuvant chemotherapy. There is current interest to move these agents into the neoadjuvant settings. While impressive improvements in survival have been demonstrated with the addition of ICI, a significant portion of patients do not respond, and the potential toxicities can be severe. In NSCLC, the only approved biomarker to predict response is the PD-L1 TPS (tumor proportion score). Studies have shown intratumoral variation in PD-L1 expression as well as discordance amongst antibodies used to assess PD-L1 expression^7,17,18^ which impacts its utility.

There has been significant interest in evaluating other biomarkers to predict response to ICI. The TMB (tumor mutational burden), which is a measure of the total number of mutations in a specimen, has been of particular interest given the hypothesis that tumors with a high TMB have a significant neoantigen load to prime activated T cells. Indeed, high TMB has been shown to be a tumor-agnostic predictor of response to checkpoint inhibition^19^ as well as improved survival with ICI^20^. However, the significance and utility of TMB in NSCLC has been less clear. As a biomarker, there is also a lack of consensus on the definition of what is high TMB (i.e. studies have used 10mut/MB^21^, 20 mut/MB^19^, among others). The MHC I (major histocompatibility complex I) genotype, thought to represent the immune system’s ability to present antigens, has also shown promise as a biomarker though work on this is earlier and has not been specifically evaluated in the clinical setting^22^. Other biomarkers that have been studied include the neutrophil-to-lymphocyte ratio^23^, and PD-L1 expression on different circulating cells i.e. platelets^24^ and antigen presenting cells^25^. Again, these putative biomarkers have not yet been explored clinically in a prospective fashion and it remains unclear whether they are predictive and/or prognostic. The focus of ongoing studies is predictors of response. It is equally important to understand determinants of non-response.

The non-neoplastic constituents of the TME play a critical role in tumor initiation and metastasis as well as response to therapy^26^. Galon et al.^10^ were one of the first groups that showed that not only the phenotype of cell mattered, but also the location of these cells. In their work on colorectal cancer, higher immune cell densities in combination with location in the tumor center AND invasive margin was associated with the longest overall survival. Previously published work from Dr. Frankel’s lab focused on refining spatial characterization of the colorectal cancer TME using methods described in this manuscript^13^ and showed that increased engagement of CTLs with ECs was associated with improved overall survival. Recently, Johnson et al.^27^ performed mfIHC on resected lung adenocarcinomas and showed that patients whose tumors harbored a high expression of MHCII in the TME had longer overall survival. We sought to explore the predictive value of TME spatial characterization in patients with metastatic NSCLC who received immune checkpoint inhibitors and evaluate factors associated with non-response.

Our study revealed that tumor PD-L1 expression, cytotoxic T cell engagement with helper T cells and cytotoxic T cell engagement with tumor cells were the three most significant factors impacting response to ICI. Combined, these three factors describe a state of heightened endogenous immune reactivity—a primed tumor microenvironment for ICIs to exert their effect. Importantly, when none of these pro-inflammatory factors was present in our cohort, there were no responses to checkpoint inhibition. Equally remarkable was that when spatial markers were present in the absence of PD-L1 expression, response to therapy was still present in those with enhanced CTL engagement highlighting the utility identifying additional patients for potential treatment. Instead of focusing on biomarkers that can predict response to ICI, it is equally important to consider biomarkers that predict non-response. Patients whose tumors exhibit this biomarker should not be exposed to the potential toxicities and costs of ICI given lack of anti-tumor benefit.

A search of clinicaltrials.gov shows there are nearly 100 active clinical trials involving immunotherapy for patients with metastatic NSCLC. These studies are examining various immune activating strategies such as combining ICI with chemotherapy or tyrosine kinase inhibitors, adding radiation, blocking other inhibitory pathways, or stimulating activating pathways. Our study suggests that spatial cellular relationships may provide a surrogate measure of endogenous immune reactivity as it related to treatment response. It would be of great interest to examine whether any of these immune activating strategies can enhance CTL engagement with tumor cells, APCs and HTLs and thereby bolster the response to PD-1/PD-L1 ICI. The tissue specimens in our study could come from any site—the primary lung, metastatic lymph nodes, or distant metastatic sites. Inter- and intratumoral heterogeneity of PD-L1 expression in well documented^28^; it is not known whether this heterogeneity also exists when assessing engagement in the TME. Though the numbers are small, we did have fourteen patients who had tissue collected at different time points and their TME was analyzed. When we looked specifically at the spatial immune score for these patients (Supplemental Figure 2), we did not see significant variance in the scores. This suggests that perhaps the spatial immune score is less subject to heterogeneity than PD-L1 alone. In several patients where the scores conflict with respect to ICI responsiveness, the contribution of each score to the overall predictability is unknown. Some scores were derived from robust tissue samples made up of multiple images, whereas some scores were generated from limited specimen. Deeper analysis of a cohort of patients with multiple samples across space and time and the predictive impact of each spatial immune score is worthy of further investigation.

With advancements in tissue imaging technology, multiplex immunofluorescent IHC, as we have described, is possible for routine analysis. mfIHC allows for evaluation of the interaction and engagement between the tumor cells and the components of the immune system as well as among the various constituents of the immune system itself. A metanalysis and systematic analysis of biomarker modalities across multiple disease types yielded seven publications focused on mfIHC^29^. To the best of our knowledge, our study is the first to report the use of mfIHC exclusively in NSCLC and also the first to report not only on quantification of the different cellular constituents, but a formal evaluation of intracellular engagement and their impact on response to ICI. We believe that our findings lend itself to additional translational studies that will enable us to better understand these interactions and their relationship to immunotherapy responsiveness which may lead to more focused use of ICI to improve the outcomes of our patients with this devastating disease.

Limitations of our study include its retrospective nature as well as changes in the treatment paradigm of NSCLC with ICI. All patients in our cohort received ICI in the second line or beyond; currently pembrolizumab (PD-1 inhibitor) is approved as first line therapy either as a single agent or in combination with chemotherapy for mNSCLC depending on PD-L1 TPS. Nivolumab (PD-1 inhibitor) in combination with ipilimumab (CTLA-4 inhibitor) is also approved in this setting. Therefore, we would need to not only validate our findings in a separate cohort of pre-treated patients, but also in patients who are receiving ICI (+/− chemotherapy) as their first line of treatment. Our study also did not stipulate that the tissue used was the biopsy immediately obtained prior to initiation of immunotherapy. Some of these biopsies were obtained at the time of diagnosis (at surgical resection) or immediately prior to systemic chemotherapy at the time of diagnosis of metastatic disease. Because the TME is dynamic and changes in response to therapy, further work is needed to confirm the validity of our findings based on timing of the tissue biopsy in relationship to ICI. Finally, we did not specifically interrogate the predictive ability of the spatial immune score to ICI versus chemotherapy. Our patients were a heavily pretreated cohort, with a range of 1–5 previous chemotherapies and the response to each specific chemotherapy regimen was not available. We did break down the spatial immune score by prior number of chemotherapy treatments (Supplemental Figure 3). The variance of scores were similar across number of lines of prior chemotherapy suggesting that the spatial immune score was not dependent on chemotherapy. Evaluating this more specifically in future studies is also worthy of consideration.

## Conclusions

Our study expands on current knowledge of the contribution of the non-neoplastic constituents of the TME and how their spatial interactions have both prognostic and predictive significance. Specifically, we show that our spatial immune score, which comprises of CTL-HTL and CTL-EC engagement and EC PD-L1 expression better predicted non-response to checkpoint inhibitors in mNSCLC compared to EC PD-L1 alone. More importantly, we also showed that in the absence of PD-L1 expression, response to ICI therapy still occurred in tumors with enhanced CTL-engagement. This score is worthy of further evaluation in a larger cohort and prospectively; our works suggests that spatial engagement should be factored when considering patients with mNSCLC for checkpoint inhibitors because it can help more clearly identify the tumors that respond or not respond.

## Supplementary Information

Supplementary Figures.

Supplementary Tables.

## Data Availability

The datasets generated during and/or analysed during the current study are available from the corresponding author on reasonable request.
