# Supplementary material for: Cellular engagement and interaction in the tumor microenvironment predict non-response to PD-1/PD-L1 inhibitors in metastatic non-small cell lung cancer
Source: Sci Rep. 2022 May 31;12:9054. doi: 10.1038/s41598-022-13236-8 (PMC9156701; doi:10.1038/s41598-022-13236-8)
Supplement: Supplementary file 1 — Supplementary Figures. [file 41598_2022_13236_MOESM1_ESM.pdf]

Supplemental Figure 1

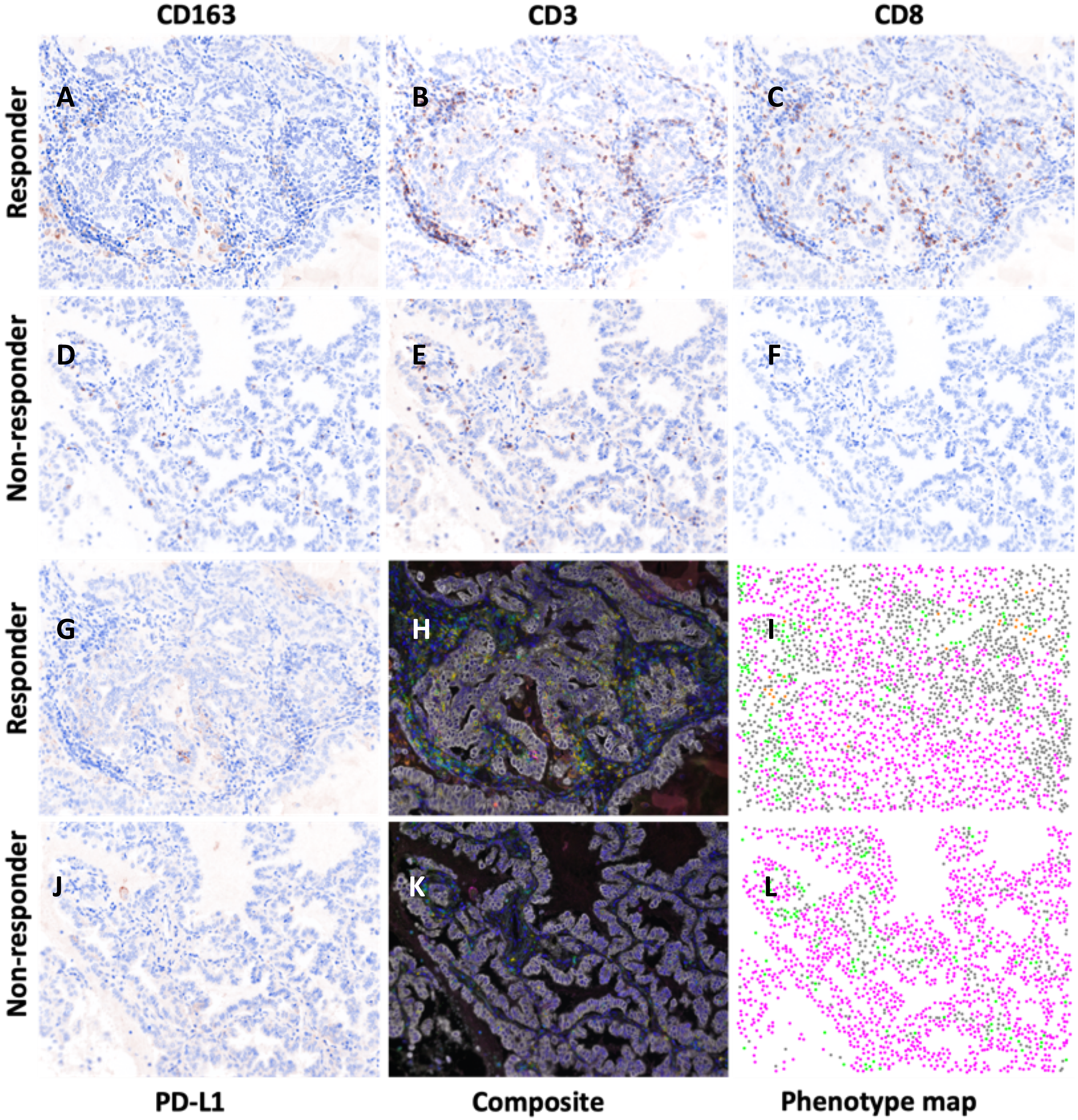

Supplemental Figure 1. shows a series of images of primary lung tissue obtained from a responder (Supp figures 1A-C, 1G-I) and a non-responder (Supp figures 1D-F, 1J-L) to immunotherapy. These images are representative and are not quantitative, but some general patterns are observed. Staining for CD163, a marker that distinguishes traditional antigen presenting cells (macrophages/dendritic cells) revealed significant cellular infiltration in responders that was largely absent in non-responders (Supp figures 1A, 1D). Similarly, responders had a greater infiltration of CD3+ and particularly CD8+ T cells (Supp figures 1B-C), whereas there was little to no CD8 staining in non-responders (Supp figures 1E-F). There also appears to be greater PD-L1 staining in responders with little in non-responders (Supp figures 1G, 1J). By employing mfIHC, multiple antigens can be probed and used to phenotype cells, preserving spatial orientation (Supp figures 1H-K; tumor cells (white), cytotoxic T cells (yellow), helper T cells (green), APC (orange), PD-L1 expression (magenta)). Phenotypic maps were generated for each image based on the x,y coordinate and multi-antigen expression pattern of each cell (Supp figure 1-L; tumor cells (pink), T cells (green), APCs (orange), unclassified cells (grey)) and used to quantify spatial relationships.

Supplemental Figure 2

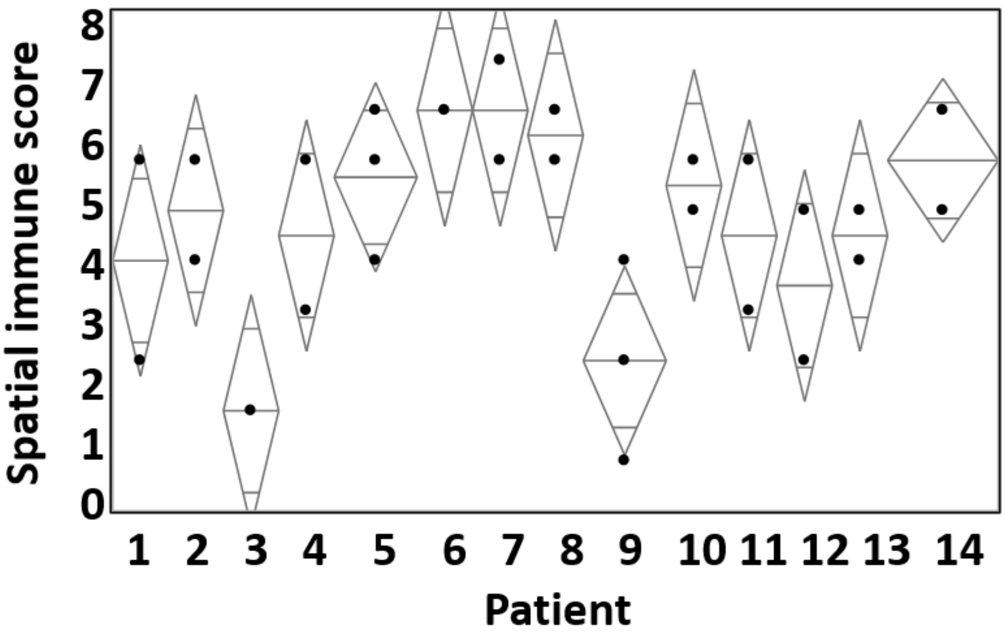

Supplemental Figure 2. Fourteen patients had more than one tissue sample, obtained at different time points, prior to ICI therapy. Each dot represents the spatial immune score of one sample. In general, there was not significant variance between scores that would have resulted in a change of prediction of ICI response.

Supplemental Figure 3

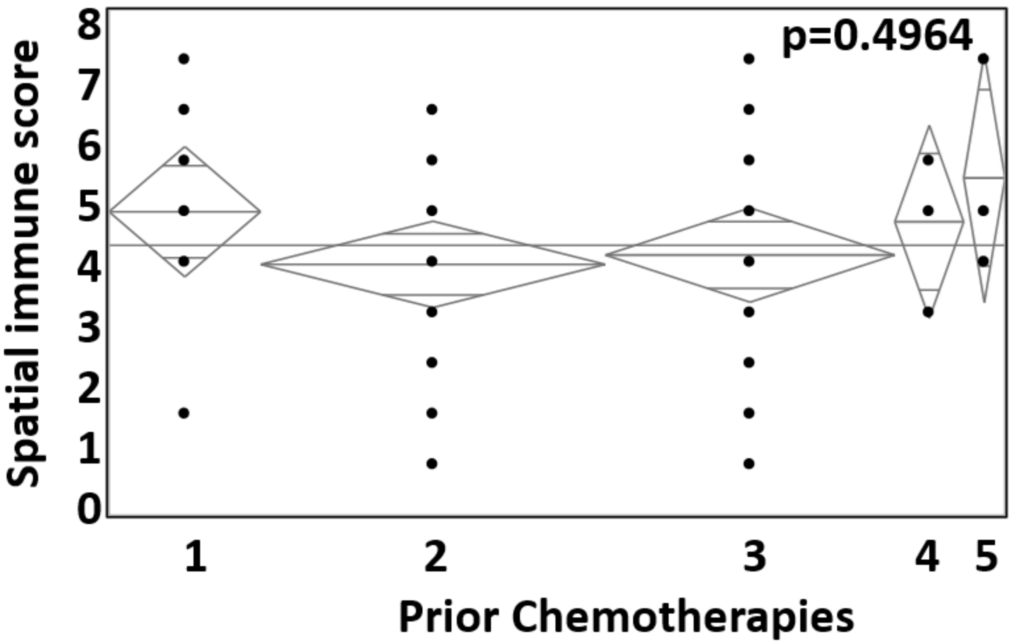

Supplemental Figure 3. The spatial immune scores were evaluated based on number of prior chemotherapy treatments. Each dot represents the score from one sample. While there was a range of scores per line of prior chemotherapy, there was no difference across the lines of prior therapy.
