# Supplementary material for: Cellular engagement and interaction in the tumor microenvironment predict non-response to PD-1/PD-L1 inhibitors in metastatic non-small cell lung cancer
Source: Sci Rep. 2022 May 31;12:9054. doi: 10.1038/s41598-022-13236-8 (PMC9156701; doi:10.1038/s41598-022-13236-8)
Supplement: Supplementary file 2 — Supplementary Tables. [file 41598_2022_13236_MOESM2_ESM.docx]

Supplemental Table 1: Antibodies and dilutions used for mfIHC

| **Primary Antibody/Antigen** | **Company-**  **Catalogue #** | **Dilution** | **Secondary**  **Antibody** | **Company-**  **Catalogue #** | **OPAL TSA^TM^**  **Fluorophore** |
| --- | --- | --- | --- | --- | --- |
| CD3 | Dako-A0452 | 1:400 | Opal Polymer® | ARH1A01EA | 520 |
| CD8 | SpringBio-M5390 | 1:400 | Opal Polymer® | ARH1A01EA | 570 |
| CD163 | Leica-NCL-L-CD163 | 1:400 | Opal Polymer® | ARH1A01EA | 650 |
| FoxP3 | CST-12653 | 1:400 | Opal Polymer® | ARH1A01EA | 620 |
| Pancytokeratin | Dako-M3515 | 1:500 | Opal Polymer® | ARH1A01EA | 690 |
| PD-L1 | CST-13684 | 1:200 | Opal Polymer® | ARH1A01EA | 540 |

Supplemental Table 2: Patient characteristics

| Characteristic |  |
| --- | --- |
| Age at diagnosis (median, range) | 65.5 (39-79) |
| Sex (n, %) |  |
| Male | 23 (44.2%) |
| Female | 29 (55.8%) |
| Smoking history (n, %) |  |
| Current | 4 (7.7%) |
| Former | 39 (75%) |
| Never | 9 (17.3%) |
| Histology (n, %) |  |
| Adenocarcinoma | 39 (75%) |
| Squamous cell carcinoma | 11 (21.2%) |
| Other | 2 (3.8%) |
| Lines of previous therapy |  |
| Mode (%), range | 1 (38.5%), [0-5] |
| Immunotherapy (n, %) |  |
| Atezolizumab | 25 (48.1%) |
| Nivolumab | 16 (30.8%) |
| Pembrolizumab | 11 (21.2%) |
